# What Factors Are Associated with Positive Effects of Dog Ownership in Families with Children with Autism Spectrum Disorder? The Development of the Lincoln Autism Pet Dog Impact Scale

**DOI:** 10.1371/journal.pone.0149736

**Published:** 2016-02-19

**Authors:** Sophie Susannah Hall, Hannah F. Wright, Daniel Simon Mills

**Affiliations:** University of Lincoln, School of Life Sciences, Lincoln, Lincolnshire, United Kingdom; Lyon Neuroscience Research Center, FRANCE

## Abstract

Scientific literature exploring the value of assistance dogs to children with autism spectrum disorder (ASD) is rapidly emerging. However, there is comparably less literature reporting the effects of pet (as opposed to assistance) dogs to these children. In particular, there are no known validated scales which assess how children may alter their behaviours in the presence of the dog, to evaluate the efficacy of pet dogs to these families. Additionally, given the highly individualised nature of ASD it is likely that some children and families gain more benefits from dog ownership than others, yet no research has reported the effect of individual differences. This pilot study reports the development of a 28-item scale based on the perceived impact of a pet dog on a child with autism by parents (Lincoln Autism Pet Dog Impact Scale—LAPDIS). The scale is comprised of three mathematically derived factors: Adaptability, Social Skills and Conflict Management. We assessed how individual differences (aspects) may be associated with scores on these three factors. Family Aspects and Dog Aspects were not significantly associated with ratings on the three factors, but Child Aspects (including: contact with horses, child age, disability level and language abilities) were related to impact of the dog on all factors. Training Aspects were related to scores on Social Skills (formal training with children with ASD and dogs and attendance at PAWS workshops run by Dogs for Good). These results suggest that individual differences associated with the child and the training approach may be important considerations for a positive impact from dog ownership on families with children with ASD. Differences in family features and the dog may not be so important, but may be worthy of further investigations given the early stage of development in this field.

## Introduction

Autism spectrum disorder (ASD) is a heterogeneous condition defined by the DSM-5 as a person experiencing persistent difficulties in social interaction, in a range of contexts, and as showing restricted, repetitive behaviours (APA 2013). These problems must have been evident in early childhood, cause significant impairment in functioning and not be explainable by intellectual disorders or developmental delays (DSM-5, APA 2013). As recognised by the term ‘spectrum’ ASD is highly complex and individualised, often co-morbid with a range of other developmental disorders (e.g. [1,2]) which can create unique challenges in developing effective treatment programmes. Therefore, it is important that therapy programmes are dynamic, being adaptable to the needs of an individual at a specific time and location.

After diagnosis clinicians may inform parents on a disparate range of numerous potential treatment programmes [3,4]. However, it is recognised that many of the existing interventions lack a strong scientific evidence base (e.g. [4,5]) which can lead to confusion for parents during this stressful time, and the adoption of inappropriate therapies. Although undertaking a clinical therapy which proves ineffective may be stressful and costly for the child and the family, the therapy can be stopped at any point. In contrast, acquiring a pet dog should be viewed as a life-long commitment, not something that can be terminated if the expectations are not meeting the reality. Therefore, it is imperative that families have good quality information available to them about the potential impact of a dog and what to expect when deciding whether or not to acquire one. Although there is increasing information available on the effects of trained dogs used in Animal Assisted Interventions (AAI), including Animal Assisted Therapy (AAT), Animal Assisted Activities (AAA) and animal assistance programs (e.g., [6– 9]) there is little literature available on the effects of pet dogs as an autism therapy. Indeed, the evidence base in the area of AAI in general is constrained by a lack of high quality studies and there is a clear need for investment in well-designed large scale clinical trials [10,11].

Nonetheless, despite the criticisms of AAI research there is growing interest and evidence to suggest that dogs can benefit children with autism in a number of ways. For instance, studies show that dogs may prime children with autism for therapy by increasing positive engagement with the therapist [12]. Dogs have also been shown to increase patient interaction and communication, and decreasing problem behaviours and stress (e.g., [6,7,13]). Moving away from the controlled environment of the therapy room, evidence indicates that when trained assistance dogs are placed in the home benefits are observed in terms of increased child safety, outdoor access, enhancement of communication and social interaction with other people and reduced child anxiety [14,15]. Many of these benefits may not be related to the specific training that the dogs have received, but rather are incidental results of the presence of the dog. Although the existing literature supporting this hypothesis is sparse, a study by [16] reports an increase in pro-social behaviours in children with autism upon acquisition of a pet (dog, cat, small furry animal), as measured by parent telephone interviews using the Autism Diagnostic Interview-Revised (ADI-R). Similarly, qualitative interviews with parents revealed that children with ASD showed more social behaviours, and less restrictive, repetitive behaviour patterns when interacting with a companion animal [17]. Additionally, interview data collected from 70 parents identified that parents generally believed their pet dog to be beneficial to their child with autism, encouraging interactions, play behaviours, companionship and responsibility [18]. These findings were partly supported by parents’ responses on standardised scales including, the Social Skills Rating Scale—which showed that children with a dog had significantly greater social skills than children without a dog, and the Companion Animal Bonding Scale—which suggested that children were highly attached to their dogs [19]. However, parents also perceive some barriers to dog ownership, such as time and cost constraints [18] as well as conflict between parents and siblings on how to best care for the dog and division of responsibilities [20]. These latter issues are not likely to be encountered during AAI, and represent potential negative factors associated specifically with pet dog therapy, as opposed to trained dog therapy, which are important to consider.

Recent studies have highlighted how the acquisition of a pet dog can bring significant improvements to parent-carers of children with autism. Using standardised assessments of parenting stress [21] and family functioning [22] significant improvements were observed in families who acquired a pet dog, compared to families who did not acquire a pet dog, over the first year of ownership. However, these studies concentrated on the potential benefits of pet dogs to the family unit rather than the child with autism.

A problem with investigating child effects is selecting the appropriate measurement to employ. Given the often limited vocabulary, communication and cooperation skills associated with autism it is challenging to collect data from the child without using observational techniques. Observational methods are subject to bias both from the participant, in terms of altered normal behaviours with the presence of a researcher/recording device, and from the researcher in the coding of behaviours. As such researchers often rely on parental reports. Given the early stage of enquiry of this developing field, exploring the effects of pet (as opposed to assistance) dogs, there are no specific scales designed to assess the effects of dog companionship on behaviours of children with ASD. Existing studies have utilised general psychological scales to measure the effects of pet dogs on children (Social Skills Rating Scale–[18]) and their parents (Parenting Stress Index Short Form–[21]; The Brief Family Functioning General Scale–[22]). A barrier in preventing the development of specific scales to measure the dependent variables of dog ownership in children with autism has been the lack of research identifying how pet dogs may affect these children. Ethical considerations prevent mass testing on items which may not be relevant to the development of the field, therefore it is important that any scale items are informed by in depth qualitative information, administered to a sample size which would provide sufficient data to explore the scale without over recruiting, and are subject to appropriate tests to explore the psychometric properties of the test.

Although the literature on the value of trained assistance dogs is growing there is a clear need to develop our understanding of how pet dogs may benefit children with ASD and to develop suitable methods for assessing these effects. Here, we report on the development of a scale to assess the effects of pet dogs on children with autism and provide the first attempt to identify the individual circumstances in which dog ownership may prove beneficial to children with autism.

The aims of this pilot, validation study were to (i) develop items to use in a scale to assess the effects of dog companionship on behaviours of children with ASD, (ii) explore the construct of this scale by documenting it’s psychometric properties, (iii) evaluate the individualised effects of pet dogs on children with ASD, to define the circumstances in which pet dog ownership may prove most effective.

## Method

### Initial Item Development

The survey items were developed from analysis of the responses of 20 semi-structured interviews with parents with children with ASD who were dog owners. Parents were recruited from a convenience sample of the UK population via Dogs for Good Parents Autism Workshops and Support (PAWS) network. The PAWS program involves a series of three workshops that educate parents about dog behaviour, welfare, and training, whist advising on the suitability of, and integration of pet dogs into families with children with ASD. Families also receive ongoing support following the workshops, including access to a range of resources such as fact sheets, videos and telephone support. In addition postings on websites and social networks related to Dogs for Good and the National Autistic Society (NAS), and word of mouth were used to increase the number of participants. Further details of this sample and the procedures used are published [20]). The interview questions were initially compiled following a review of the existing literature, and then circulated to the project advisory group for additional input and discussion. The project advisory group was made up of twelve members including autism professionals, psychology professionals, veterinary professionals, assistance dog professionals, academics and parents of children with ASD who own a family pet dog. Feedback from the group resulted in an interview schedule addressing specific areas associated with dog ownership in families with children with autism. Based on these procedures 28 separate items were developed for the survey (see Table 1). Eleven of the items were designed to be negatively worded to avoid set responses. A five point scale was used for responding to each item: 1 = Strongly Agree, 2 = Agree, 3 = Strongly Disagree, 4 = Strongly Disagree. Participants were also required to respond to a series of demographic questions. For purpose of the analysis the demographic questions were grouped into four sections; Family Aspects, Child Aspects, Dog Aspects and Training Aspects (see Tables 2–5).

**Table 1:** Items in the Survey.

No.	Item
***Adaptability***	
1	My child is more likely to tolerate changes in his/her normal routine if he/she is with the dog
2	My child shows more independence in his/her self-care behaviours if he/she is with the dog (e.g., is better at washing, or dressing when the dog is there)
4	My child seems happier in him/herself when he/she is with the dog
6	My child is more affectionate towards human family members when he/she is with the dog
7	My child is less afraid of other dogs when he/she is with our dog
9	My child is less likely to have tantrums or meltdowns when he/she is with the dog
10	My child is able to pay attention on an imposed task (i.e., something you have asked them to do) when he/she is with the dog
12	My child is less likely to engage in repetitive behaviours (e.g., hand flapping, pacing) when he/she is with the dog
15	My child is more willing to engage in new activities or experiences if he/she is with the dog
16	My child is more likely to show empathy for another family member (e.g., instinctively feel sad or them, rather than being prompted or instructed by another) when he/she is with the dog
18	My child shows more use of imagination when engaging in play with other people when he/she is with the dog (e.g., will follow the lead of others and engage in the ‘spirit’ of the game)
19	My child is more willing to go out for a walk with other family member when he/she is with the dog
21	My child shows more independence within the home if he/she is with dog (e.g., would be more likely to off into another room away from family member if the dog is with him/her)
22	As a parent I feel I am more able to have time to myself when my child is with the dog
24	Family activities are more enjoyable when the child is with the dog
25	My child recovers from tantrums or meltdowns more quickly when the dog is there
28	My child is more likely to show imagination in his/her play (e.g., it’s not always the same pattern of play or game) when he/she is with the dog
***Social Skills***	
8	My child is less likely to engage in an appropriate social interaction with a new or unfamiliar person if he she is with the dog
11	My child is less likely to communicate his/her immediate needs to a family member (either verbally or non-verbally) when he/she is with the dog
13	We have less flexibility in our routines when the child is with the dog
14	My child is less likely to engage in a social interaction with another family member when he/she is with the dog
17	My child is more hesitant to interact with other dogs when he/she is out with our dog
20	My child is less likely to communicate his/her feelings to another family member when he/she is with the dog
23	My child is less likely to co-operate with another family member if he/she is with the dog
***Conflict Management***	
3	My child is more likely to get into conflict with his/her siblings when he/she is with the dog
5	We are less able to get out of the house to complete routine tasks when the child is with the dog
26	We have more family arguments and disagreements when the child is with the dog
27	My child shows more running off or bolting behaviour when he/she is with the dog

**Table 2:** Membership to Group Based on Family Aspects; Total n and Percentage.

Family Aspects	Factor 1: Adaptability (A)		Factor 2: Social Skills (SS)		Factor 3: Conflict Management (CM)	
	High Scores (Lower A with dog)	Low Scores (Higher A with dog)	High Scores (Greater SS with dog)	Low Scores (Fewer SS with dog)	High Scores (Better CM with dog)	Low Scores (Reduced CM with dog)
	*Group 1*	*Group 2*	*Group 1*	*Group 2*	*Group 1*	*Group 2*
***Parent Gender***						
Male	7 (4%)	2 (7%)	7 (4%)	2 (7%)	7 (4%)	2 (7%)
Female	160 (96%)	28 (93%)	160 (96%)	28 (93%)	160 (96%)	28 (93%)
***Parent Age***						
Under 25 years	3 (2%)	0 (0%)	3 (2%)	0 (0%)	3 (2%)	0 (0%)
25–35 years	34 (20%)	8 (27%)	34 (20%)	8 (27%)	34 (20%)	8 (27%)
36–50 years	120 (72%)	20 (67%)	120 (72%)	20 (67%)	120 (72%)	20 (67%)
40–51 plus years	8 (5%)	2 (7%)	8 (5%)	2 (7%)	8 (5%)	2 (7%)
Prefer not to say	2 (1%)	0 (0%)	2 (1%)	0 (0%)	2 (1%)	0 (0%)
***Parent Country of Residence***						
United Kingdom	163 (98%)	0 (0%)	163 (98%)	0 (0%)	163 (98%)	0 (0%)
Australia	1 (0.5%)	0 (0%)	1 (0.5%)	0 (0%)	1 (0.5%)	0 (0%)
Belgium	1 (0.5%)	0 (0%)	1 (0.5%)	0 (0%)	1 (0.5%)	0 (0%)
Canada	2 (1%)	0 (0%)	2 (1%)	0 (0%)	2 (1%)	0 (0%)
European Union	0 (0%)	2 (7%)	0 (0%)	2 (7%)	0 (0%)	2 (7%)
Ireland	0 (0%)	7 (23%)	0 (0%)	7 (23%)	0 (0%)	7 (23%)
Netherlands	0 (0%)	9 (30%)	0 (0%)	9 (30%)	0 (0%)	9 (30%)
Philippines	0 (0%)	1 (3%)	0 (0%)	1 (3%)	0 (0%)	1 (3%)
United States	0 (0%)	11 (37%)	0 (0%)	11 (37%)	0 (0%)	11 (37%)
***Parents in Household***						
One	37 (22%)	3 (10%)	37 (22%)	3 (10%)	37 (22%)	3 (10%)
Two	130 (78%)	27 (90%)	130 (78%)	27 (90%)	130 (78%)	27 (90%)
***Regular Support from Unpaid Family and Friends***						
No	18 (11%)	6 (20%)	18 (11%)	6 (20%)	18 (11%)	6 (20%)
Yes	149 (89%)	24 (80%)	149 (89%)	24 (80%)	149 (89%)	24 (80%)
***Regular Support from Paid Carers***						
No	19 (11%)	7 (23%)	19 (11%)	7 (23%)	19 (11%)	7 (23%)
Yes	148 (89%)	23 (77%)	148 (89%)	23 (77%)	148 (89%)	23 (77%)
***Total Number of People Living in the Home***						
1 person	5 (3%)	0 (0%)	5 (3%)	0 (0%)	5 (3%)	0 (0%)
2 people	27 (16%)	4 (13%)	27 (16%)	4 (13%)	27 (16%)	4 (13%)
3 people	81 (49%)	18 (60%)	81 (49%)	18 (60%)	81 (49%)	18 (60%)
4 people	32 (19%)	6 (20%)	32 (19%)	6 (20%)	32 (19%)	6 (20%)
5 people	19 (11%)	1 (3%)	19 (11%)	1 (3%)	19 (11%)	1 (3%)
Over 5	3 (2%)	1 (3%)	3 (2%)	1 (3%)	3 (2%)	1 (3%)
***Total Number of Children***						
1 child	9 (6%)	1 (3%)	9 (6%)	1 (3%)	9 (6%)	1 (3%)
2 children	89 (53%)	21 (70%)	89 (53%)	21 (70%)	89 (53%)	21 (70%)
3 children	40 (24%)	6 (20%)	40 (24%)	6 (20%)	40 (24%)	6 (20%)
4 children	22 (13%)	7%)	22 (13%)	7%)	22 (13%)	7%)
5 children	5 (3%)	0 (0%)	5 (3%)	0 (0%)	5 (3%)	0 (0%)
Over 5 children	2 (2%)	0 (0%)	2 (2%)	0 (0%)	2 (2%)	0 (0%)
***Number of Children with ASD Diagnosis***						
1 child	141 (84%)	27 (90%)	141 (84%)	27 (90%)	141 (84%)	27 (90%)
2 children	23 (14%)	3 (10%)	23 (14%)	3 (10%)	23 (14%)	3 (10%)
3 children	3 (2%)	0 (0%)	3 (2%)	0 (0%)	3 (2%)	0 (0%)
***Family Income***						
Up to £10,000	16 (10%)	1 (3%)	16 (10%)	1 (3%)	16 (10%)	1 (3%)
£10–20,000	31 (19%)	1 (3%)	31 (19%)	1 (3%)	31 (19%)	1 (3%)
£20–40,000	60 (36%)	6 (20%)	60 (36%)	6 (20%)	60 (36%)	6 (20%)
£40–60,000	26 (16%)	1 (3%)	26 (16%)	1 (3%)	26 (16%)	1 (3%)
£50–60,000 plus	9 (5%)	1 (3%)	9 (5%)	1 (3%)	9 (5%)	1 (3%)
Prefer not say	25 (15%)	20 (67%)	25 (15%)	20 (67%)	25 (15%)	20 (67%)
***Age Child Acquired Dog***						
1–4 years	59 (35%)	14 (47%)	59 (35%)	14 (47%)	59 (35%)	14 (47%)
5–8 years	54 (32%)	9 (30%)	54 (32%)	9 (30%)	54 (32%)	9 (30%)
9–12 years	43 (26%)	6 (20%)	43 (26%)	6 (20%)	43 (26%)	6 (20%)
13–16 years	11 (7%)	1 (3%)	11 (7%)	1 (3%)	11 (7%)	1 (3%)
***Previous Dog Ownership***						
Never owned a dog before	26 (16%)	6 (20%)	26 (16%)	6 (20%)	26 (16%)	6 (20%)
At least 1 parent had a dog as a child, but not as an adult	58 (35%)	5 (17%)	58 (35%)	5 (17%)	58 (35%)	5 (17%)
At least 1 parent had a dog as an adult before, but outside of this family	20 (12%)	8 (27%)	20 (12%)	8 (27%)	20 (12%)	8 (27%)
This family has had at least 1 dog before	63 (38%)	11 (37%)	63 (38%)	11 (37%)	63 (38%)	11 (37%)

**Table 3:** Membership to Group Based on Child Aspects; Total n and Percentage.

Child Aspects	Factor 1: Adaptability (A)		Factor 2: Social Skills (SS)		Factor 3: Conflict Management (CM)	
	High Scores (Lower A with dog)	Low Scores (Higher A with dog)	High Scores (Greater SS with dog)	Low Scores (Fewer SS with dog)	High Scores (Better CM with dog)	Low Scores (Reduced CM with dog)
	*Group 1*	*Group 2*	*Group 2*	*Group 1*	*Group 2*	*Group 1*
***Child Age***	7.04±3.3yrs	7.95±3.9yrs	8.01±3.8yrs	6.64±3.5yrs	11.7±2.1yrs	5.41±2.3yrs
***Child Diagnosis***						
Autism	32 (43%)	45 (37%)	51 (37%)	26 (45%)	23 (18%)	54 (78%)
ASD	28 (38%)	36 (29%)	44 (32%)	20 (34%)	21 (16%)	43 (62%)
Asperger’s	12 (16%)	39 (31%)	41 (29%)	10 (17%)	25 (20%)	26 (38%)
Other	2 (3%)	3 (2%)	3 (2%)	2 (3%)	0 (0%)	5 (7%)
***Child Disability Level***						
Severe	8 (11%)	6 (5%)	10 (7%)	4 (7%)	2 (3%)	12 (9%)
Moderate	22 (30%)	38 (31%)	36 (26%)	24 (41%)	17 (25%)	43 (34%)
High functioning	44 (59%)	79 (64%)	93 (67%)	30 (52%)	50 (72%)	73 (57%)
***Child Language Ability***						
No speech	4 (5%)	4 (3%)	5 (4%)	3 (5%)	0 (0%)	8 (6%)
Single words/gestures	7 (9%)	3 (2%)	6 (4%)	4 (7%)	1 (1%)	9 (7%)
Simple phrases	12 (16%)	15 (12%)	17 (12%)	10 (17%)	5 (7%)	22 (17%)
Sentences	51 (69%)	101 (82%)	111 (80%)	41 (71%)	63 (91%)	89 (70%)
***Child Understanding of Language***						
No understanding	1 (1%)	0 (0%)	1 (1%)	0 (0%)	0 (0%)	1 (1%)
Basic understanding	7 (9%)	3 (2%)	7 (5%)	3 (5%)	0 (0%)	10 (8%)
Moderate (simple sentences)	20 (27%)	34 (28%)	31 (22%)	23 (40%)	17 (25%)	37 (29%)
Good (everyday speech)	46 (62%)	86 (70%)	100 (72%)	32 (55%)	52 (75%)	80 (63%)
***Child has Other Conditions***						
Yes	30 (41%)	67 (54%)	69 (50%)	28 (48%)	37 (54%)	60 (47%)
No	44 (59%)	56 (46%)	70 (50%)	30 (52%)	32 (46%)	68 (53%)
***Child has Lived with Dogs Previously***						
Yes	23 (31%)	37 (30%)	47 (34%)	13 (22%)	27 (39%)	33 (26%)
No	51 (69%)	86 (70%)	92 (66%)	45 (78%)	42 (61%)	95 (74%)
***Child has Regular Contact with Other Dogs***						
Yes	35 (47%)	47 (38%)	61 (44%)	21 (36%)	28 (41%)	54 (42%)
No	39 (53%)	76 (62%)	78 (66%)	37 (64%)	41 (59%)	74 (58%)
***Child has Regular Contact with Horses***						
Yes	3 (4%)	25 (20%)	22 (16%)	6 (10%)	10 (14%)	18 (14%)
No	71 (96%)	98 (80%)	117 (84%)	52 (90%)	59 (86%)	100 (78%)
**Child has Regular Contact with Other Animals**						
No	35 (47%)	66 (54%)	71 (51%)	30 (52%)	36 (52%)	65 (51%)
Yes	39 (53%)	57 (46%)	68 (49%)	28 (48%)	33 (48%)	63 (49%)

**Table 4:** Membership to Group Based on Dog Aspects; Total n and Percentage.

Dog Aspects	Factor 1: Adaptability (A)		Factor 2: Social Skills (SS)		Factor 3: Conflict Management (CM)	
	High Scores (Lower A with dog)	Low Scores (Higher A with dog)	High Scores (Greater SS with dog)	Low Scores (Poorer SS with dog)	High Scores (Better CM with dog)	Low Scores (Reduced CM with dog)
	*Group 1*	*Group 2*	*Group 2*	*Group 1*	*Group 2*	*Group 1*
***Dog Age***						
Less than 6 months	0 (0%)	1 (0.5%)	0 (0%)	1 (0.6%)	0 (0%)	1 (0.7%)
6 months– 1 years	1 (4%)	16 (9%)	3 (9%)	14 (9%)	0 (0%)	17 (12%)
1–3 years	11 (44%)	81 (47%)	20 (59%)	72 (44%)	0 (0%)	92 (64%)
3–6 years	8 (32%)	40 (13%)	7 (21%)	41 (25%)	18 (34%)	30 (21%)
6–10 years	4 (16%)	23 (13%)	4 (12%)	23 (14%)	24 (45%)	3 (2%)
10 years and over	1 (4%)	11 (6%)	0 (0%)	12 (7%)	11 (21%)	1 (0.7%)
***Dog Breed***						
Pure breed	12 (48%)	106 (62%)	21 (62%)	97 (60%)	30 (57%)	88 (61%)
Cross breed	11 (44%)	61 (35%)	13 (38%)	59 (36%)	22 (42%)	50 (35%)
Unknown	2 (8%)	5 (3%)	0 (0%)	7 (4%)	1 (9%)	6 (4%)
***Length of Time Owning the Dog***						
Less than 6 months	0 (0%)	0 (0%)	0 (0%)	0 (0%)	0 (0%)	0 (0%)
6 months– 1 year	3 (12%)	34 (20%)	5 (15%)	32 (20%)	0 (0%)	37 (26%)
1–2 years	6 (24%)	52 (30%)	13 (38%)	45 (28%)	0 (0%)	58 (40%)
2–3 years	6 (24%)	22 (13%)	7 (21%)	21 (13%)	0 (0%)	28 (19%)
3–4 years	1 (4%)	20 (12%)	2 (6%)	19 (12%)	4 (8%)	17 (12%)
4–5 years	3 (12%)	8 (5%)	4 (12%)	7 (4%)	8 (15%)	3 (2%)
5–6 years	1 (4%)	11 (6%)	1 (3%)	11 (7%)	11 (21%)	1 (0.6%)
6–7 years	0 (0%)	9 (5%)	1 (3%)	8 (5%)	9 (17%)	0 (0%)
8 years plus	5 (20%)	16 (9%)	1 (3%)	20 (12%)	21 (40%)	0 (0%)
***Sex of the Dog***						
Male	17 (68%)	83 (48%)	22 (65%)	78 (48%)	25 (47%)	75 (52%)
Female	8 (32%)	89 (52%)	12 (35%)	85 (52%)	28 (53%)	69 (48%)
***Dog Neutered***						
Yes	17 (68%)	116 (67%)	24 (71%)	109 (67%)	44 (83%)	89 (62%)
No	8 (32%)	56 (33%)	20 (59%)	54 (33%)	9 (17%)	55 (38%)
***Where the Dog was Sourced***						
Other	1 (4%)	6 (3%)	2 (6%)	5 (3%)	1 (2%)	6 (4%)
Professional breeder	9 (36%)	68 (40%)	13 (38%)	64 (39%)	21 (40%)	56 (39%)
Private / one-off breeder	8 (32%)	53 (31%)	10 (29%)	51 (31%)	12 (23%)	49 (34%)
Rescue centre	5 (20%)	25 (15%)	5 (15%)	25 (15%)	17 (32%)	13 (9%)
Private rehome	2 (8%)	18 (10%)	4 (12%)	16 (10%)	2 (4%)	18 (13%)
Failed guide/assistance dog	0 (0%)	2 (1%)	0 (0%)	2 (1%)	0 (0%)	2 (1%)
***Age of Dog on Arrival***						
Less than 2 months	8 (32%)	51 (30%)	5 (15%)	54 (33%)	14 (26%)	45 (31%)
2–4 months	10 (40%)	83 (48%)	18 (53%)	75 (46%)	22 (42%)	71 (49%)
5–12 months	1 (4%)	16 (9%)	5 (15%)	12 (7%)	6 (11%)	11 (8%)
12–36 months	5 (20%)	13 (8%)	4 (12%)	14 (9%)	6 (11%)	12 (8%)
Over 36 months (3 years)	1 (4%)	9 (5%)	3 (9%)	8 (5%)	5 (9%)	5 (3%)

**Table 5:** Membership to Group Based on Training Aspects; Total n and Percentage.

Training Aspects	Factor 1: Adaptability (A)		Factor 2: Social Skills (SS)		Factor 3: Conflict Management (CM)	
	High Scores (Lower A with dog)	Low Scores (Higher A with dog)	High Scores (Greater SS with dog)	Low Scores (Poorer SS with dog)	High Scores (Better CM with dog)	Low Scores (Reduced CM with dog)
	*Group 1*	*Group 2*	*Group 2*	*Group 1*	*Group 2*	*Group 1*
***Training Approach***						
Reward based	41 (71%)	89 (64%)	80 (70%)	50 (61%)	61 (69%)	69 (63%)
Correction based	1 (2%)	2 (1%)	1 (0.8%)	2 (2%)	2 (2%)	1 (1%)
Mixture of reward and correction based	16 (28%)	48 (35%)	34 (30%)	30 (37%)	25 (28%)	39 (36%)
***Attended Formal Training—Dog and Children with ASD***						
No	47 (81%)	113 (81%)	88 (77%)	72 (88%)	71 (81%)	89 (82%)
Yes	11 (19%)	26 (19%)	27 (23%)	10 (12%)	17 (19%)	20 (18%)
***Attended Formal Training—Dog and Children with ASD—PAWS Workshops***						
No	47 (81%)	114 (82%)	88 (77%)	73 (89%)	71 (81%)	90 (83%)
Yes	11 (19%)	25 (18%)	27 (23%)	9 (11%)	17 (19%)	19 (17%)
***Gain Knowledge on Dog Behaviour/Training from the TV*?**						
No	42 (72%)	99 (71%)	81 (70%)	60 (73%)	59 (67%)	82 (75%)
Yes	16 (28%)	40 (29%)	34 (30%)	22 (27%)	29 (33%)	27 (25%)
***Gain Knowledge on Dog Behaviour/Training from the Internet***						
No	28 (48%)	81 (58%)	63 (55%)	46 (56%)	51 (58%)	58 (53%)
Yes	30 (52%)	58 (42%)	52 (45%)	36 (44%)	37 (42%)	51 (47%)
***Gain Knowledge on Dog Behaviour/Training from the Dog Breeder***						
No	51 (88%)	120 (86%)	100 (87%)	71 (87%)	77 (88%)	94 (86%)
Yes	7 (12%)	19 (14%)	15 (13%)	11 (13%)	11 (12%)	15 (14%)
***Gain Knowledge on Dog Behaviour/Training from Friends/Family***						
No	39 (67%)	89 (64%)	71 (62%)	57 (70%)	57 (65%)	71 (65%)
Yes	19 (33%)	50 (36%)	44 (38%)	25 (30%)	31 (35%)	38 (35%)
***Gain Knowledge on Dog Behaviour/Training from Books/Magazines***						
No	37 (64%)	88 (63%)	72 (63%)	53 (65%)	55 (63%)	70 (64%)
Yes	21 (36%)	51 (37%)	43 (37%)	29 (35%)	33 (37%)	39 (36%)
***Gain Knowledge on Dog Behaviour/Training from Dog Trainers/Behaviourists***						
No	38 (66%)	96 (69%)	78 (68%)	56 (68%)	63 (72%)	71 (65%)
Yes	20 (34%)	43 (31%)	37 (32%)	26 (32%)	25 (28%)	38 (35%)
***Gain Knowledge on Dog Behaviour/Training from PAWS Workshops/ Dogs For Good Team***						
No	49 (84%)	114 (82%)	91 (79%)	72 (88%)	71 (81%)	92 (84%)
Yes	9 (16%)	25 (18%)	24 (81%)	10 (12%)	17 (19%)	17 (16%)

### Participants

Informed written consent was obtained from each participant, and all procedures complied with British Psychological Society “Code of Ethics and Conduct”, and with the World Medical Association Helsinki Declaration as revised in October 2008. The ethical committee in the School of Life Sciences, University of Lincoln approved the study. All respondents to the survey were required to own one dog, which was not an assistance dog and have a child with ASD. Participants confirmed this at the start of the survey. A total of 347 participants responded to the online survey. Those with missing data were removed, leaving a total of 197 responses in the analysis. We chose to remove incomplete responses because we cannot rule out that where participants chose not to respond to items this was because they were presenting a bias response. For instance, families may choose to only respond to items in which the dog has a positive effect to their child. The demographic details of the sample are displayed in Tables 2–5. The categories defining the child’s disability level and language ability were determined based on consultation with clinical experts and are comparable to those used in previous studies [21,22]. Parents were asked to select which category best represented their child.

### Design and Procedure

The items were entered into Survey Monkey in a random order (order selected using a computerised randomisation program). The order in which each item was presented in listed in Table 1. Participants were sent a link to the online survey. Instructions were presented on the screen, asking the participants to respond to each question as honestly as possible and that there were no right or wrong answers. After confirming consent questions were presented in a list format, with 8 questions presented per page. Responses were saved automatically. The survey was online for 12 months, from June 2012 to June 2013. Data was collated and analysed once the survey had closed.

### Statistical Analysis

The purpose of this pilot study was to assess whether the questions selected from the item development process where appropriate for future investigations (outside of this present study) to explore the test re-rest reliability of the scale and its predictive validity. Therefore, the first step of the analysis was to determine the factor structure of the scales. To allow any factor structure to emerge we used Exploratory Factor Analysis (EFA). The number of variables (*n* = 28) to participants (*n* = 374) ratio exceeded the 5 to 1 ratio (with a minimum number of participants, *n* > 150) which exceeds the standard recommendation for EFA [23,24]. Inspection of the Scree plot was used to determine the number of factors to extract, before Principal Component Analysis (PCA) was undertaken using a promax rotation, as we expected the factors to be correlated, with delta set to 0 and specifying the number of factors in the solution, based on the results of the factor analysis. Meaningful loadings were assessed using the criteria of 0.32 = poor, 0.45 = fair, 0.55 = good, 0.63 = very good, 0.71 = excellent [25,26]. Cronbach's alpha [27] coefficients were used to assess internal reliability of factors with interpretative criteria of α ≤ 0.6, < 0.7 = 'acceptable' and α > .70 = 'good' [28,29].

To explore how four sources of individual difference (Demographics: Family Aspects, Child Aspects, Dog Aspects and Training Aspects) were related to individual factor scores we conducted a hierarchical cluster analysis. Because this is an initial stage validation study of the scale we were interested in discovering how demographic factors were related to scores on the factors in the scale, rather than controlling for the effect of demographic factors, as this may artificially remove important individual differences relating to the benefits of pet dogs. By using Wards method and squared Euclidean distance as the similarity measure we examined maximum differences between the clusters (as opposed to associations). Analyses were conducted separately for each demographic aspect across each of the factors, resulting in multiple analyses. Prior to conducting the cluster analysis we explored the optimum number of clusters in the data sets by visually inspecting dendrogram plots and the coefficient change scores. We specified two clusters in the analysis to split the data set into high and low responders, in order to understand which factors are important in determining successful (improved) living with a dog. To assess if the two clusters were significantly different from each other independent t-tests (for continuous data) or chi-square tests (for categorical data) were conducted.

## Results

Factor analysis showed a clear 3 factor solution to the data. All 28 items showed ‘good’ loading at equal to or above 0.55 on one of the factors, with the average loading being .73 for Factor 1, .65 for Factor 2 and .61 for Factor 3. Factor 1 was comprised of 17 positively worded questions, which were themed around ‘Adaptability’ and are presented in Table 1. Factor 1 (Adaptability) accounted for 34% of the variance. The second factor was comprised of 7 negatively worded relating to social interactions and therefore Factor 2 was named Social Skills, which accounted for 14% of the variance. The third factor consisted of 4 negatively worded questions relating to ‘Conflict Management’, this factor (Conflict Management) accounted for 5% of the variance. The correlations between the 3 factors were Adaptability and Social Skills = .20, Adaptability and Conflict Management = -.22, and Social Skills and Conflict Management = .17; indicating that the factors measured separate elements of the effects of pet dogs on children with autism. Cronbach's alpha for the three factor were all good: Adaptability α = .93, Social Skills α = .77 and Conflict Management α = .71.

One hundred and ninety seven cases (participants) responding to the demographic questions and scale items were entered into the cluster analysis. Since analyses were conducted separately for each demographic aspect across each of the three factors, there were 12 separate analyses. Inspection of the dendrogram showed a large variation within the data. However, in all analyses two over-arching groups could be determined. The results are reported separately for each of the demographic aspects. See Tables 2–5 for the mean number of participants (and percent) in each group. Scores on Factor 1, Adaptability, were comprised solely of positively worded questions, therefore a score 1 (Strongly Agree) was a positive effect and a score of 5 (Strongly Disagree) was a negative effect. As such, lower total scores reflect greater adaptability in the presence of the dog. Conversely, Factor 2 and Factor 3 were comprised only of negatively worded questions, therefore a score of 1 (Strongly Agree) was a negative effect and a score of 5 (Strongly Disagree) was a positive effect. As such, higher total scores reflect greater social skills and improved conflict management in the presence of the dog.

### Family Aspects

See Table 2 for detailed information on group membership relating Family Aspects.

#### Factor 1: Adaptability

There was no significant difference between the two groups in adaptability (*t*(195) = .740, *p* = .460). The two groups differed significantly based on country of residence and family income. Group 1 was comprised of more parents from the UK, Australia, Belgium and Canada. Group 2 was comprised of more parents living elsewhere in the European Union, Philippines and the United States (χ² = 197.00, df = 8, *p* = .000). More parents in Group 1 selected their income group (χ² = 39.45, df = 5, *p* = .000). The groups were not significantly different based on any other family aspects.

#### Factor 2: Social Skills

There was no significant difference between the two groups in social skills (*t*(195) = 1.49, *p* = .137). The two groups differed significantly on country of residence and family income, in the same way as described for Factor 1 (country of residence χ² = 197.00, df = 8, *p* = .000, income group χ² = 39.45, df = 5, *p* = .000). The groups were not significantly different based on any other family aspects.

#### Factor 3: Conflict Management

There was no significant difference between the two groups based on social skills (*t*(195) = 0.69, *p* = .489). As with Factor 1 and Factor 2, the two groups were only significantly different from each other based on country of residence and family income (tests of statistical inference were identical to that reported in Factor and Factor 2).

The groups that emerged from the cluster analysis were not significantly different based on their scores of Adaptability, Social Skills and Conflict Management with the dog, and showed the same grouping based on family aspects. This suggests that family aspects do not significantly affect parent’s perceptions of behaviour impact of the dog.

### Child Aspects

See Table 3 for detailed information on group membership relating to Child Aspects.

#### Factor 1: Adaptability

Two groups emerged which significantly differed in their scores of adaptability (*t*(195) = 17.86, *p* = .000). The smaller group (Group 1: n = 74), showed less adaptability in the presence of the dog compared to the larger group (Group 2: n = 123). Significantly more children in Group 2 had contact with horses than children in Group 1 (χ² = 10.03, df = 1, *p* = .002), implying increased adaptability in the presence of the dog was associated with children who had more regular contact with horses.

#### Factor 2: Social Skills

The two groups were significantly different based on their scores of social skills (*t*(195) = 14.25, *p* = .000). Group 1 (n = 58) showed fewer social skills in the presence of the dog in comparison to Group 2 (n = 139). Group 2 was comprised of significantly older children in comparison to Group 1 (*t*(195) = 2.36, *p* = .01), implying increased social skills in the presence of the dog, was associated with having an older child.

#### Factor 3: Conflict Management

The two groups were significantly different based on their scores of conflict management (*t*(195) = 3.56, *p* = .000). Group 1 (n = 128), showed reduced conflict management in the presence of the dog in comparison to Group 2 (n = 69). The two groups were significantly different from each other based on child age, child diagnosis and child language abilities. Children in Group 2 were significantly older than children in Group 1 (*t*(195) = 18.58, *p* = .000), had less severe disability on the autism spectrum (χ² = 8.12, df = 3, *p* = .04), and had better language abilities (χ² = 13.05, df = 3, *p* = .005).

The results suggest that better conflict management from the presence of the dog is associated with children who are older, who are on the higher functioning scale of autism spectrum disorder and who have better abilities.

### Dog Aspects

See Table 4 for detailed information on group membership relating to Dog Aspects.

#### Factor 1: Adaptability

The two groups were significantly different in their scores of adaptability (*t*(195) = 15.24, *p* = .000). Group 1 (n = 25), demonstrated lower adaptability in the presence of the dog compared to Group 2 (n = 172). The two groups did not significantly differ from each other based on individual differences on the dog aspects. However, there was a near statistically significant effect of dog sex (χ² = 3.40, df = 1, *p* = .06), with people in Group 2 owning more female dogs than male dogs. The results suggest that increased adaptability in the presence of the dog might be associated with owning a female dog, but this effect of dog gender, if real, is small.

#### Factor 2: Social Skills

The two groups scored significantly differently on social skills (*t*(195) = 12.05, *p* = .000). Group 1 (n = 163), showed fewer social skills in the presence of a dog in comparison to Group 2 (n = 34). The groups did not significantly differ based on individual differences in dog aspects, indicating that children’s social skills in the presence of a dog are not significantly affected by dog aspects.

#### Factor 3: Conflict Management

The two groups were not significantly different based on their scores of conflict management, although the *p* value approached significance (*t*(195) = 1.82, *p* = .07), with Group 1 demonstrating reduced conflict management in the presence of the dog in comparison to Group 2, but we do not consider this effect reliable given the multiple testing undertaken. The groups were significantly different from each other based on dog age, how long the dog was owned for, whether the dog was neutered or not and where the dog came from. Families in Group 2 owned older dogs than families in Group 1 (χ² = 121.57, df = 5, *p* = .000). More families in Group 2 had owned the dog for a longer period of time (χ² = 164.77, df = 7, *p* = .000), and had a dog that was neutered (χ² = 7.94, df = 1, *p* = .005). The groups also differed based on where the dog was obtained from (χ² = 19.35, df = 5, *p* = .02).

### Training Aspects

See Table 5 for detailed information on group membership based on Training Aspects.

#### Factor 1: Adaptability

There was a significant difference between the two groups based on adaptability (*t*(195) = 18.21, *p* = .000). Group 1 (n = 58) showed lower adaptability in the presence of the dog in comparison to Group 2 (n = 139). The groups did not significantly differ on any of the training aspects, indicating that high and low responses were not associated with individual differences in training aspects.

#### Factor 2: Social Skill

There was a significant difference between the two groups based on social skills (*t*(195) = 15.93, *p* = .000). Group 1 (n = 82) showed fewer social skills in the presence of the dog in comparison to Group 2 (n = 115). The two groups were significantly different from each other based on whether or not they had attended formal dog training with children with ASD and whether they had attended PAWS workshops. More parents in Group 2 had attended formal training (χ² = 3.99, df = 1, *p* = .04), and PAWS workshops (χ² = 5.01, df = 1, *p* = .02). The results suggest that children who show better social skills in the presence of the dogs are those whose parents have attended formal training with dogs and children with ASD and PAWS workshops.

#### Factor 3: Conflict Management

There was a significant difference between the two groups based on conflict management (*t*(195) = 16.70, *p* = .000). Group 1 (n = 109) was made up of more members who showed reduced conflict management in the presence of the dog in comparison to Group 2 (n = 88). The groups did not significantly differ on any of the training aspects, suggesting that high and low responses in conflict management were not associated with individual differences in training.

## Discussion

With the aim of piloting the development of an assessment scale for evaluating the impact of pet dog ownership on children with ASD we designed a 28 item survey. The items were developed based on discussions with our advisory group and the results of semi-structured interview with parents [20]. With the aim of documenting the psychometric properties of this scale we conducted PCA and internal reliability analysis (alpha coefficients). The results showed that the scale was comprised of 3 factors, which were themed around Adaptability, Social Skills and Conflict Management. All the factors demonstrated good internal reliability, and were reported by parents on the advisory group as having good comprehensibility. In order to identify which types of individual difference influence impact of the dog on the child, we identified significant differences between high and low responders to the clustered items based on Family, Child, Dog, and Training Aspects. The results suggest that Family Aspects are not significantly associated with perceived impact of the dog on any of the three factors identified. Child Aspects did affect experience with dog, on all the factors (adaptability, social skills and conflict management), highlighting important individual child differences in the therapeutic effect of dog ownership in these families. Dog Aspects had little impact, and this might be due to preselection of the dog by the family to determine the type of dog that appears to fit best with the family, indicating that the effect is a general “dog” effect, assuming successful integration into the family. Training Aspects were related to social skills, in particular attendance at a workshop aimed at setting realistic expectations and maximising the potential of the family dog. We discuss the results in relation to each of the three child related factors in order to collate the potential effects for practitioners and parents who may be interested in exploring strategies to improve child functioning in these areas.

### Adaptability

Rigidity in behaviours and thought patterns is a hallmark symptom associated with autism [30], as well as impacting on the behaviours of the individual with autism these symptoms can impact on the entire family unit, reducing their ability to partake in, and enjoy, a range of different activities. Indeed, it has also been suggested that first degree relatives may also show problems with [31], although it is arguably difficult to isolate whether this is a genetic or learned trait. Evidence suggests that adaptability is important in determining effectiveness of family based autism interventions [32]. Therefore, in order to develop the potential of dog ownership as an effective tool for improving adaptability it is important that we examine under what environment (i.e., individual differences) dogs are most effective at improving adaptability.

The results indicated that adaptability (questions related to both children with autism and the family), improved when the dog was present if the child had more regular contact with horses. There was some evidence to suggest that increased adaptability was associated with owning a female as opposed to male dog, but this effect if real is small, and probably not of clinical relevance- it is perhaps more important that the family get the dog who they feel is right for them. Adaptability was not significantly associated with individual differences in family factors or training on the integration of the dog into the family. These findings suggest that perhaps, in general, exposure to social non-human species may be beneficial to children (or the family unit) who have a particular difficulty with adaptability, and that the effects of exposure to different species may be synergistic. The benefits of equine therapy to children with autism are becoming of increasing interest (e.g., the horse boy method). There is some evidence to suggest that activities with horses can benefit children with ASD, with observed improvements in children’s social behaviours and functioning [33,34], sensory processing and seeking [33,35] and attention and self-regulation behaviours [33,36]. However, these conclusions are based on small scale studies, which often lack a suitable control sample/condition. Owing to a number of differences in design and analysis it is difficult to relate studies highlighting the effects of horses to children with autism to studies reporting the effects of dogs. Nonetheless, our results suggest that in combination with dog ownership, contact with horses may help improve the adaptability of children with autism. Owing to the broad definition used in our study (regular contact with horses), it is not possible to define which aspect of contact with a horse may be important, and how this may relate to having a dog present. Furthermore, it is not possible to decipher whether contact with horses requires the child to be actively involved in equine therapy, or if it is the petting or presence of a horse that is important. Possible speculations include that these animals help to reduce children’s repetitive behaviours by providing a stimulating distraction and breaking obsessive cycles. It is also possible that these animals help to calm and reassure the child which increases their confidence in completing new tasks [36]. Although the anxiety reducing effects of horses are not well studied, the anxiety reducing effects of the presence of a dog is of growing scientific interest, and deserves further investigation. Many reports indicate that dogs can bring a calming effect to human arousal [37–39,13], but some evidence is contrary to this indicating that dogs have an excitatory effect [40], or no effect [41]. A further possibility is that the presence of an animal may help to improve adaptability by increasing family freedom and ensuring child safety. Research shows that trained assistance dogs help families to safely engage in new activities and public outings by providing an anchor point and a calming influence [14]. Therefore, it is possible that the presence of a dog supports the child to engage with horses.

### Social Skills

There is a well-documented history of problems in social skills and functioning in individual with autism (e.g., [42–45]. Despite the prevalence of social skill deficits in autism spectrum disorder, few children have access to effective social skills programming [46]. Indeed, results from systematic reviews and meta-analysis indicate little support for current interventions [47,48]. Social skills have a wide reaching impact on outcomes including, academic performance, social failure, anxiety, depression, suicide and substance abuse [49–52]. Therefore, it is important that we define the individual circumstances in which dog ownership may be most effective in improving social skills.

The results highlight that children who show a greater improvement in social skills in the presence of the dog are older children (child aspects), and those who have had a parent attend PAWS workshops and formal training with dogs and children with ASD. There were no significant differences between high and low responders based on family aspects or dog aspects. These findings suggest that if children have a particular problem with social skills functioning parent’s may want to consider acquiring a dog once their child gets older (approximately 8 years seems a reasonable recommendation, given the current data). However, the effect of age on social skills is unlikely to be unique to being in the presence of the dog. Given that social skills show a gradual maturation process it is quite possible that improved social skills with age could be found in relation to any intervention/condition. It may prove important to note that those who attended formal training, through PAWS workshops (organised by Dogs for Good) and other training showed better social skills. This could suggest that the improved social skills associated with acquiring a trained assistance dog [14] may not be due to the incidental nature of the dog, but instead may reflect the importance of training both the dog and the handler (parent) to maximise the benefits of presence of the dog. This is in contrast to observations reported by [16], who stated an increase in pro-social behaviours in children with autism upon acquisition of a pet. However, [16] explored the arrival of a pet, which may bring about novelty effects, as opposed to longer term living with a pet. Additionally, [16] examined the effects of acquiring a ‘pet’ as opposed to specifically a pet dog. Whilst the effect of training highlights the importance of organisations such as PAWS it should also be pointed out that it could be that parents who have attended PAWS workshops are more alert to detecting changes in their child’s social behaviours, which may have contributed to the effect observed here. Future studies could investigate the impact of educating parents on detecting small changes in their child’s behaviour in a group of parents who have attended PAWS workshops and in a matched control group.

### Conflict Management

Autism spectrum disorder is associated with frequent child tantrums [53] and there is a relationship between family conflict and severity of ASD symptomology [54]. It is thought that children with ASD may be particularly sensitive to conflict due to their problems with sensory sensitivity and perspective [54]. Therefore, an important aspect in developing a family therapy for ASD would be to consider under what circumstances conflict management is improved. Our analysis indicates that conflict management is improved in the presence of the dog if the child is older, has better language abilities, has less severe disabilities and has owned a dog before. Dog factors seem to have little effect, but, given our results, might deserve further investigation in future. No significant effects were observed with family and or training aspects. The child aspects surrounding age, language and disability level are not surprising, and as with the previous results discussed, are unlikely to be unique to owning a dog. It would be expected that as children get older conflict management is improved, possibly through parents learning the most effective way to avoid, or solve any conflicts as they arise. Furthermore, previous research has identified a relationship between family conflict and ASD symptomology [54], supporting the result here that families whose children are higher functioning and show better languages abilities show better conflict management. The possible effect of owning a dog for longer and owning an older dog, might indicate that experience with being a dog owner may be important in effectively resolving conflict behaviours in the presence of the dog. In any case helping owners to gain the necessary expertise associated with successfully owning a dog for a longer time, should always be encouraged.

Overall, these results indicate that positive outcomes surrounding adaptability, social skills and conflict management are associated with a range of individual related factors. Whilst acquiring a pet dog may bring a range of benefits to many families living with a child with ASD, not all families are likely to experience the same improvements and to the same degree. Our results provide two particularly important contributions. Firstly, we have developed a useful scale (Lincoln Autism Pet Dog Impact Scale—LAPDIS) for assessing the impact of the presence of a pet dog in children with ASD. This is the first scale of its kind, targeted on the specific effects reported by a large sample of parents internationally. Secondly, we describe the initial disentangling of the individualised circumstances which might influence the efficacy of dog ownership within families with a child with ASD, identify important controls for more mechanistically focussed research in this field in future. It is clear that the dog is not a simple intervention, and it is likely to have multiple effects that vary between families [55]. Understanding these factors is important if families are to receive the best quality advice from their clinical advisors. We should perhaps point out, that these data clearly relate to families who want a dog and so a dog should not be prescribed to those who are not willing to make the additional commitment required for its successful integration into the family. Additionally, whilst the data presented here shows that pet dogs can be a positive addition to family life and highlights the need for further research to explore the impact of pet dogs as well as trained assistance dogs, we do not claim that pet dogs can replace the important role played by trained assistance dogs [56]. Indeed, whereas assistance dogs are chosen specifically for their temperament (e.g. calmness) and characteristics (e.g. stable enough to prevent a child from bolting when on a dual-lead) to support children with ASD pet dogs come in many shapes and sizes. Therefore future research should further explore how dog characteristics may affect the perceived benefit of dog ownership and compare child functioning in families who own a trained assistance dog with those who own a pet dog.

However, we stress that further research is needed to test the hypotheses generated by this study. Further validation of the LAPDIS scale is required to assess test re-test reliability and predictive validity. Additionally, it is clear that further research is needed to assess a wider range of individual differences and to conduct controlled comparisons exploring child and family responses to situations based on the individual differences identified here. Furthermore, due to survey design it was not practical to make independent assessments of the child’s ASD symptoms. Therefore, we relied on parent’s confirmation that their child had received a clinically confirmed diagnosis of ASD, as with our earlier studies in this area [21,22]. Given that parents had to give up 40–45 minutes of their time, with no tangible reward, there was little incentive for parents to be dis-honest in reporting their child’s diagnosis. Nonetheless, future studies could ensure that parent’s completed standardised checklists of symptoms as a control to improve upon this present design.

This study documents how sores on the three LAPDIS scale factors (Adaptability, Social Skills, and Conflict Management) are associated with individual differences in the child, and the training undertaken and possibly the dog, but not the family. The results further highlight important individual differences which may affect the value of pet dogs to families with children with autism which should be considered when deciding whether to acquire a dog and what might be reasonably expected given the families’ circumstances.

## References

[1] Kohane IS, McMurry A, Weber G, McFadden D, Rappaport L, Kunkel L, et al The co-morbidity burden of children and young adults with autism spectrum disorders. PLoS One. 2012; 7(4):e33224 10.1371/journal.pone.0033224 22511918 PMC3325235

[2] Mukaddes NM, Fateh R. High rates of psychiatric co-morbidity in individuals with Asperger's disorder. World J Biol Psychiatry. 2010;11(2–2):486–492. 19347776 10.1080/15622970902789130

[3] Autism Speaks (2010). How is Autism Treated? Accessed online http://www.autismspeaks.org/sites/default/files/documents/100-day-kit/treatment_version_2_0.pdf, last accessed 20/08/2014.

[4] Hamburg MA, Collins FS. The path to personalized medicine. N Engl J Med. 2010;363(4): 301–304. 10.1056/NEJMp1006304 20551152

[5] Simpson R, de Boer-Ott SR, Griswold D, Griswold DE, Myles BS, Byrd SE. et al Autism spectrum disorders: Interventions and treatments for children and youth. Corwin Press; 2004.

[6] Berry A, Borgi M, Francia N, Alleva E, Cirulli F Use of assistance and therapy dogs for children with autism spectrum disorders: A critical review of the current evidence. J Altern Complement Medicine. 2013;19(2): 73–80. 10.1089/acm.2011.0835 22978246

[7] O’Haire ME. Animal-assisted intervention for autism spectrum disorder: A systematic literature review. J Autism Dev Disord. 2013;43(7):1606–1622. 10.1007/s10803-012-1707-5 23124442

[8] Prothmann A, Ettrich C, Prothmann S. Preference for, and responsiveness to, people, dogs and objects in children with autism. Anthrozoös. 2009;22(2): 161–171.

[9] Solomon O. What a dog can do: Children with autism and therapy dogs in social interaction. Ethos. 2010;38: 143–166.

[10] Mills D, Hall S. Animal-assisted interventions: making better use of the human-animal bond. Vet Rec. 2014;174(11): 269–273. 10.1136/vr.g1929 24627508

[11] O'Haire M. Companion animals and human health: Benefits, challenges, and the road ahead. J Vet Behav. 2010;5(5): 226–234.

[12] Silva K, Correia R, Lima M, Magalhães A, de Sousa L. Can dogs prime autistic children for therapy? Evidence from a single case study. J Altern Complement Med. 2011;17(7): 655–659. 10.1089/acm.2010.0436 21689015

[13] Viau R, Arsenault-Lapierre G, Fecteau S, Champagne N, Walker CD, Lupien S. Effect of service dogs on salivary cortisol secretion in autistic children. Psychoneuroendocrinology. 2010;35(8): 1187–1193. 10.1016/j.psyneuen.2010.02.004 20189722

[14] Burrows KE, Adams CL, Spiers J. Sentinels of safety: Service dogs ensure safety and enhance freedom and well-being for families with autistic children. Qual Health Res. 2008;18(12): 1642–1649. 10.1177/1049732308327088 18955467

[15] Redefer LA, Goodman JF. Brief report: Pet-facilitated therapy with autistic children. J Autism Dev Disord. 1989;19(3): 461–467. 2793790 10.1007/BF02212943

[16] Grandgeorge M, Tordjman S, Lazartigues A, Lemonnier E, Deleau M, Hausberger M. Does pet arrival trigger prosocial behaviors in individuals with autism. PloS One. 2012;7(8): e41739 10.1371/journal.pone.0041739 22870246 PMC3411605

[17] Byström KM, Lundqvist Persson CA. The meaning of companion animals for children and adolescents with autism: The parents' perspective. Anthrozoos. 2015;28(2): 263–275.

[18] Carlisle GK. Pet dog ownership decisions for parents of children with Autism Spectrum Disorder. J Paediatr Nurs. 2014;29(2): 114–123. 10.1016/j.pedn.2013.09.005 24183985

[19] Carlisle GK. The social skills and attachment to dogs of children with Autism Spectrum Disorder. J Autism Devel Disord. 2015;45(5): 1137–1145. 25308197 10.1007/s10803-014-2267-7

[20] Wright H, Hall S, Hames A, Hardiman J, Mills R, Mills D. (In press). Perceived impact of pet dogs on children with Autism Spectrum Disorders (ASD) and their families: Expectations versus reality. Hum Anim Interactions Bull.

[21] Wright H, Hall S, Hames A, Hardiman J, Mills R, Mills D. Acquiring a pet dog significantly reduces stress of primary carers for children with Autism Spectrum Disorder: A prospective case control study. J Autism Devel Disord. 2015a: 1–10. 25832799 10.1007/s10803-015-2418-5 PMC4513200

[22] Wright H, Hall S, Hames A, Hardiman J, Mills R, Mills D. Pet dogs improve family functioning and reduce anxiety in children with Autism Spectrum Disorders Anthrozoos. 2015b. In press.

[23] Cattell RB. The Scientific use of Factor Analysis in Behavior and Life Sciences. New York: Plenum; 1978.

[24] Gorsuch RL. Factor Analysis. 2nd ed Hillsdale, NJ: Erlbaum; 1983.

[25] Comrey AL, Lee HB. A First Course in Factor Analysis. 2nd ed Hillsdale, NJ: Erlbaum;1992.

[26] Tabachnick BG, Fidell LS. Experimental Designs using ANOVA. Thomson/Brooks/Cole; 2007.

[27] Cronbach LJ. Coefficient alpha and the internal structure of tests. Psychometrika.1951;16(3): 297–334.

[28] Kline RB. Principles and Practices of Structural Equation Modeling. New York: Guilford; 1998.

[29] Nunnally JC. Psychometric Theory. McGraw Hill, New York, NY; 1978.

[30] American Psychiatric Association. 5th Ed Diagnostic and Statistical Manual of Mental Disorders Washington, DC: Author; 2013.

[31] Hurley RS, Losh M, Parlier M, Reznick JS, Piven J. The broad autism phenotype questionnaire. J Autism Devel Disord. 2007;37(9): 1679–1690. 17146701 10.1007/s10803-006-0299-3

[32] Baker JK, Seltzer MM, Greenberg JS. Longitudinal effects of adaptability on behavior problems and maternal depression in families of adolescents with autism. J Fam Psychol. 2011; 25(4): 601 10.1037/a0024409 21668120 PMC3987806

[33] Bass MM, Duchowny CA, Llabre MM. The effect of therapeutic horseback riding on social functioning in children with autism. J Autism Devel Disord. 2009; 39(9): 1261–1267. 19350376 10.1007/s10803-009-0734-3

[34] Lanning BA, Baier MEM, Ivey-Hatz J, Krenek N, Tubbs JD. Effects of equine assisted activities on Autism Spectrum Disorder. J Autism Devel Disord. 2014; 44(8): 1897–1907. 24526337 10.1007/s10803-014-2062-5

[35] Ward SC, Whalon K, Rusnak K, Wendell K, Paschall N. The association between therapeutic horseback riding and the social communication and sensory reactions of children with autism. J Autism Devel Disord. 2013; 43(9): 2190–2198. 23371511 10.1007/s10803-013-1773-3

[36] Gabriels RL, Agnew JA, Holt KD, Shoffner A, Zhaoxing P, Ruzzano S. et al Pilot study measuring the effects of therapeutic horseback riding on school-age children and adolescents with autism spectrum disorders. Res Autism Spect Disord. 2012; 6(2): 578–588.

[37] Beetz A, Julius H, Turner D, Kotrschal K. Effects of social support by a dog on stress modulation in male children with insecure attachment. Front Psychol. 2012; 3: 352 10.3389/fpsyg.2012.00352 23162482 PMC3498889

[38] Friedmann E, Katcher A, Thomas S, Lynch J, Messene P. Social interaction and blood pressure: influence of animal companions. J Nerv Mental Disord. 1983;171(8): 461–465. 10.1097/00005053-198308000-00002 6875529

[39] O'Haire ME, McKenzie SJ, Beck AM Slaughter V. Animals may act as social buffers: Skin conductance arousal in children with autism spectrum disorder in a social context. Dev Psychobiol. 2015; Available http://www.researchgate.net/profile/Virginia_Slaughter/publication/275525679_Animals_may_act_as_social_buffers_Skin_conductance_arousal_in_children_with_autism_spectrum_disorder_in_a_social_context_Animals_and_Autism/links/5547d8a80cf26a7bf4da9948.pdf. Accessed 27 May 2015. 10.1002/dev.21310 PMC8447904 25913902

[40] Somervill JW, Swanson AM, Robertson RL, Arnett MA, MacLin OH. Handling a dog by children with attention-deficit/hyperactivity disorder: Calming or exciting. N Am J Psychol. 2009;11(1): 111–120.

[41] Gee NR, Friedmann E, Stendahl M, Fisk A, Coglitore V. Heart rate variability during a working memory task: Does touching a dog or person affect the response? Anthrozoos. 2014; 27(4): 513–528.

[42] Attwood T. Asperger’s syndrome: A guide for parents and professionals. Philadelphia: Kingsley; 1998.

[43] Carter AS, Davis NO, Klin A, Volkmar FR. Social development in autism Handbook of Autism and Pervasive Developmental Disorders, Volume 1, Third Edition, 312–334; 2005

[44] Howlin P. The effectiveness of interventions for children with autism In Neurodevelopmental Disorders, Springer Vienna; 2005 pp. 101–119. 10.1007/3-211-31222-6_6 16355605

[45] Myles BS, Adreon DA, Hagen K, Holverstott J, Hubbard A, Smith SM, et al Life journey through autism: An educator’s guide to Asperger syndrome. Arlington, VA: Organization for Autism Research; 2005.

[46] Hume K, Bellini S, Pratt C. The usage and perceived outcomes of early intervention and early childhood programs for young children with autism spectrum disorder. Topics Early Child Spec Educ. 2005;25(4): 195.

[47] Bellini S, Peters JK, Benner L, Hopf A. A meta-analysis of school-based social skills interventions for children with autism spectrum disorders. Remedial and Special Education. 2007;28(3): 153–162.

[48] Reichow B, Steiner AM, Volkmar F. Cochrane review: social skills groups for people aged 6 to 21 with autism spectrum disorders (ASD). Evid Based Child Health. 2013;8(2): 266–315. 10.1002/ebch.1903 23877884

[49] Bellini S. The development of social anxiety in adolescents with autism spectrum disorders. Focus on Autism and Other Developmental Disabilities. 2006;21(3), 138–145.

[50] La Greca AM, Lopez N. Social anxiety among adolescents: Linkages with peer relations and friendships. J Clin Child Psychol. 1998;26:83–94. 10.1023/a:1022684520514 9634131

[51] Tantam D. Psychological disorder in adolescents and adults with Asperger syndrome. Autism. 2000;4: 47–62.

[52] Welsh M, Park RD, Widaman K, O’Neil R. Linkages between children’s social and academic competence: A longitudinal analysis. J Sch Psychol. 2001;39: 463–481.

[53] Konst MJ, Matson JL, Turygin N. Exploration of the correlation between autism spectrum disorder symptomology and tantrum behaviors. Res Autism Spectr Disord. 2013;7(9): 1068–1074.

[54] Kelly AB, Garnett MS, Attwood T, Peterson C. Autism spectrum symptomatology in children: The impact of family and peer relationships. J Abnorm Child Psychol. 2008;36(7): 1069–1081. 10.1007/s10802-008-9234-8 18437549

[55] Wright HF, Hall S, Mills DS. Additional evidence is needed to recommend acquiring a dog to families of children with Autism Spectrum Disorder: A response to Crossman and Kazdin. J Autism Dev Disord. 2015; 1–4. 26340955 10.1007/s10803-015-2548-9

[56] Burgoyne L, Dowling L, Fitzgerald A, Connolly M, Browne JP, Perry IJ. Parents’ perspectives on the value of assistance dogs for children with autism spectrum disorder: a cross-sectional study. BMJ open. 2014;4(6), e004786 10.1136/bmjopen-2014-004786 24928583 PMC4067897

